# Corpus cavernosum smooth muscle cell dysfunction and phenotype transformation are related to erectile dysfunction in prostatitis rats with chronic prostatitis/chronic pelvic pain syndrome

**DOI:** 10.1186/s12950-019-0233-z

**Published:** 2020-01-06

**Authors:** Guang-chun Wang, Tian-run Huang, Yang-yang Hu, Ke-yi Wang, Heng Shi, Lei Yin, Bo Peng

**Affiliations:** Department of Urology, Shanghai Tenth People’s Hospital, Tongji University School of Medicine, NO. 301 Yanchang Road, Shanghai, 200072 People’s Republic of China

**Keywords:** Prostatitis, Erectile dysfunction, Smooth muscle cells

## Abstract

**Background:**

The relationship between chronic prostatitis/chronic pelvic pain syndrome (CP/CPPS) and erectile dysfunction (ED) has been shown in many studies. However, the specific mechanism remains unclear. This study was to investigate the corpus cavernosum smooth muscle cell function and phenotype transformation in Experimental autoimmune prostatitis (EAP) rats.

**Methods:**

EAP was induced in rats by using prostate protein supplemented with immuneadjuvant extraction, and the max-ICP and MAP were measured. IHC and Masson staining were done to assess inflammatory infiltration and collagen deposition in the corpus cavernosum, respectively. Subsequently, normal rat and EAP rat CCSMCs were purified by tissue block implantation and differential adherence method. The oxidative stress, smooth muscle phenotype transformation, cell cycle and intracellular calcium ion transport were also evaluated.

**Results:**

The ratio of max ICP/MAP in EAP rats significantly reduced, and the TNF-α content and collagen deposition in the corpus cavernosum markedly increased as compared to healthy rats. High-purity rat CCSMCs were obtained. Oxidative stress was evident and the cGMP content decreased in the EAP rat CCSMCs. The expression of Cav1.2, IP3R1 and RyR2 increased, but the SERCA2 expression decreased in EAP rat CCSMCs, which was accompanied by increased intracellular calcium. Increased expression of OPN, collagen and KCa3.1, decreased Calponin expression and increased proportion of cells in the S phase were also observed in the EAP rat CCSMCs.

**Conclusion:**

CP causes oxidative stress and imbalance of intracellular calcium in CCSMCs and promotes CCSMCs transformation from contractile to synthetic state, which may be involved in the pathogenesis of ED.

## Introduction

Prostatitis is one of common urological diseases in men younger than 50 years and may significantly affect the quality of life in these patients [1]. Chronic prostatitis/chronic pelvic pain syndrome (CP/CPPS) is the most common type of prostatitis [2], and about 15% of men may suffer from prostatitis-related symptoms once [3]. Clinical manifestations of prostatitis are not limited to the inflammation of the prostate, and they are also accompanied with symptoms of urinary tract irritation, perineal pain and sexual dysfunction. Most symptoms of CP/CPPS can be categorized into urinary symptoms, psychosocial dysfunction, organ-specific findings, infection, neurological/systemic abnormalities and tenderness of the muscles (UPOINT) [4]. In recent years, studies focus on the sexual dysfunction in men with CP/CPPS and “Sexual dysfunction” in the UPOINT is also proposed [5, 6]. The incidence of erectile function (ED) in Chinese CP/CPPS patients is 15.0–40.5% [7, 8], and CP/CPPS patients are 3.62-fold more likely than healthy individuals to suffer from ED [9]. However, the underlying mechanism underlying the relationship between prostatitis and ED remains unclear.

Corpus cavernosum is composed of endothelial-lined sinusoids which are surrounded by fibrous tissues and smooth muscles. Corpus cavernosum smooth muscle cells (CCSMCs) account for 38.5–52.0% of total cells in the corpus cavernosum [10]. Before erection, the released nitric oxide (NO), which is produced by the endothelial nitric oxide synthase (eNOS) and neuronal nitric oxide synthase (nNOS) under physiological conditions, increases the production of cyclic guanosine monophosphate (cGMP), which then relaxes CCSMCs in the arterial wall. The increase in the arterial inflow may distend the sinusoids within the corpora cavernosa, increasing the intracavernous pressure, which results in erection. However, little is known about whether prostatitis causes ED via damaging CCSMCs.

In our previous study, a rat model of experimental autoimmune prostatitis (EAP) was established and ED [11] and cavernous endothelial cells dysfunction [12] were observed in these EAP rats. This study investigated the intracellular calcium and phenotype transformation of CCSMCs in EAP rats, which may provide evidence on the pathogenesis of ED in EAP rats.

## Materials and methods

### Establishment of EAP rat model

Male Sprague-Dawley (SD) rats, 6–8 weeks, were used in this study. The study was conducted in line with the “Guidelines for the Care and Use of Laboratory Animals” published by the National Institutes of Health and approved by the Animal Science Committee of Tongji University. EAP rats was established as previously reported [11, 12]. Ten rats were used for preparing autologous prostate tissue homogenate supernatant (PTHS). Additional 40 rats were randomly divided into EAP group and control group (20 rats per group). In the EAP group, each rat was administered with 1.0 mL of isovolumetric mixture of PTHS (20 mg/mL) and Freund’s complete adjuvant by multipoint subcutaneous injection; meanwhile, 0.5 mL of a pertussis–diphtheria–tetanus vaccine was intraperitoneally injected. In the control group, each rat was injected with isovolumetric phosphate buffered saline. After treatment at days 0, 15, and 30, the rat model of EAP was established.

### Assessment of erectile function

Rats was sacrificed at 45th day after the first immunization. At 45th day after the first immunization, the max intracavernous pressure (ICP) and the ratio of max ICP to mean systemic arterial pressure (max ICP/MAP) were determined to assess the erectile function as previously reported [11, 12]. The erectile response was elicited by electrical stimulation on the cavernous nerve and quantified by calculating the max ICP/MAP. Stimulations were performed in triplicate at 5 V and lasted for 30 s with an interval of 5 min between two stimulations.

### Inflammatory infiltration of rat prostate and corpus cavernosum

After the assessment of erectile function, rats were sacrificed. The penises and prostate were immediately collected. Some corpus cavernosum and prostate tissues were fixed overnight in 4% paraformaldehyde, and the remaining tissues were stored in liquid nitrogen for further analysis. The fixed tissues were dehydrated in 70% ethanol, embedded in paraffin and then sectioned. After hematoxylin staining for 15 min, the sections were rinsed with running water for 1–3 s, treated with 1% hydrochloric acid ethanol for 1 min and the washed in double distilled water for 10–30 s, followed by 0.5% eosin staining for 3 min. The sections were rinsed and dehydrated.

### Immunohistochemistry

After washing thrice with PBS, 50–100 μl of normal goat serum was added to each section, followed by incubation at room temperature for 20 min. Then, the primary antibody (1: 400 sc-52,746) was added, followed by incubation for 2 h. After washing thrice with PBS, 50 μl of enhancer was added, followed by incubation for 30 min at room temperature. After washing thrice with PBS, the secondary antibody was added, followed by incubation for 30 min. Then, the sections were washed with PBS, stained with DAB and then with hematoxylin for 10 min, dehydrated and mounted. The expression of TNF-α was observed under a light microscope.

### Isolation and purification of CCSMCs

The penis was collected and washed with pre-cooled PBS containing 100 μg/mL penicillin and streptomycin to remove blood. The tunica albuginea was removed from the penile tissues under a microscope (Nikon, Model C-DSD230, Japan), and then the remaining corpus cavernosum was cut into 1 mm^3^ blocks. The tissue blocks were placed with an interval of 1 cm in a sterile flask containing 20% FBS, and then 2 ml of complete DMEM medium containing 20% FBS and 1% double antibody was added. The tissue block was placed upwards and incubated at 37 °C in an environment with 5% CO_2_. The flask was gently shaken to ensure sufficient contact of tissue blocks with culture medium. Three days later, the flask was observed under an inverted microscope, and then 8 ml of complete DMEM was added. The flasks were incubated until 80% cell confluence. The culture medium was removed, and the adherent cells were rinsed with PBS and then digested with 0.5 ml of 0.05% EDTA-containing trypsin. The serum-containing medium was added to terminate the digestion. The cell suspension was gently pipetted and then centrifuged at 200 g for 10 min. The supernatant was removed, and the remaining cells were harvested. Complete DMEM was added to suspended cells, and the cell suspension was transferred into a new flask, followed by inoculation at 37 °C in an environment with 5% CO_2_ for 30 min. The suspended cells were transferred to a new culture flask, and above-mentioned operation was repeated 2 times.

### Immunofluorescence staining

Purified CCSMCs were cultured on coverslips in the Millicell (Millipore, PEZGS 0816, USA). When 80–90% confluence was achieved, cells were fixed in 4% paraformaldehyde for 30 min, permeabilized with Triton X-100 for 20 min, blocked with 5% BSA for 1 h, incubated with primary antibodies (anti-Desmin (1:400, ab32362), anti-α-SMA (1:400, ab124964), anti-CD31 (1:400, ab119339) and anti-CD90/Thy1 (1:200, ab92574) at 4 °C overnight and then treated with fluorescein-conjugated secondary antibodies at room temperature for 2 h. Subsequently, the cell nuclei were stained with 4, 6-diamidino-2-phenylindole. The sections were observed under a confocal microscope (ZEISS LSM700).

### Measurement of oxidative stress and cGMP level

SOD activity and MDA content were detected to assess the oxidative stress. The SOD activity, MDA content and cGMP level were measured with commercial kits (Nanjing Jiancheng Bioengineering Company, China).

### Q-PCR assay

Total RNA was extracted from tissues using TRIzol, and the RNA concentration was determined. The mRNA was reversely transcribed into cDNA which was used as a template for qPCR. The reaction mixture included 10 μL 2× Real-time PCR Master Mix, 2 μL of each primer and 7 μL of EPC water. The reaction was done as follows: pre-denaturation at 95 °C for 5 min, denaturation at 40 °C for 15 s, annealing at 60 °C for 20 s and elongation at 72 °C for 40 s. The primer sequences are shown in Table 1.

**Table 1:** The primer sequences used in the present study

Item	5′---------3’
OPN-F	TGGATGTCTACCAGCGAAGC
OPN-R	ACGCACTCCAGGGCTTCA
CaV1.2-F	AACAGGGCAACATCTACAA
CaV1.2-R	TCCGCAATCACATCTTCA
IP3R1-F	CAGGTGGATGGCGATGAA
IP3R1-R	TGGGCACAGGGAAGACAA
RYR2-F	AGTGATTGGCAGCAGGTC
RYR2-R	GGTGTCTGGGATGTTTAGG
Kca3.1-F	GGGACTCTTCACGCTTCG
Kca3.1-R	CACCTTCAGGCTCAACCA
Calponin-F	TCTCGGCTTAGGGCATGGAT
Calponin-R	TCTATGACGCCGTGATAGCAG
SERCA2-F	TGGATTTGGACGCATTGGTC
SERCA2-R	TTTGCACTGGTACGTGTTGAT
GAPDH-F	GCAAGTTCAACGGCACAG
GAPDH-R	GCCAGTAGACTCCACGACAT

### Western blotting

Cells were rinsed with PBS and then transferred into 250 mL of ice-cold modified RIPA buffer (Shanghai Beyotime Institute of Biotechnology, China) containing protease and phosphatase inhibitors. The cell lysates were collected and transferred to EP tubes. Lysates were sonicated for 20 s (25% power, 0.5 cycles) and centrifuged at 12000 g for 30 min at 4 °C. The supernatant was then transferred to a new tube. Protein concentration was determined with the BCA assay (Shanghai Beyotime Institute of Biotechnology). Forty milligram of proteins were separated on the 10–12% SDS-PAGE gel and transferred onto PVDF membranes. The PVDF membrane was then blocked with 5% milk for 2 h at room temperature and incubated with the primary antibody overnight (anti- Cav1.2 (1:1000, ACC-003) and anti-KCa3.1 (1:200, APC-064), anti- IP3R1 (1:500, sc-271,197) and anti-SERCA2 (1:500, sc-53,010), anti-Calponin antibody (1:500, bs-0095R), anti-ryanodine receptor antibody (1: 500, bs-6305R), anti-osteopontin (OPN) antibody (1:1000, bs-0026R) and anti-Collagen antibody (1:1000, bs-10423R). After rinsing 4 times in Tris buffered saline containing 0.1% Tween 20 (TBST) (Thermo, 34,160 and 31,430) for 15 min, rabbit or mouse antibodies conjugated to horseradish peroxidase (HRP) were added, followed by incubation for 2 h in dark. Protein bands were autoradiographed and quantified with an Enhanced Chemiluminescence (ECL) system (Beyotime Institute of Biotechnology) by densitometry. GAPDH (1:1000 sc-32,233) was used as an internal reference.

### Flow cytometry assay

Intracellular calcium of CCSMCs was detected by flow cytometry. According to the manufacturer’s instructions (Fluo-3 AM Cell Membrane Permeable Calcium Fluorescent Probe), the cells were seeded in 24-well plates and maintained until 80% confluence. Then, 5 μM Fluo-3 AM was added, followed by incubation at 37 °C in an environment with 5% CO_2_ for 1 h in dark. The cells were digested with EDTA-free trypsin, then centrifuged at 200 g for 10 min, rinsed twice with calcium-magnesium-free HBSS, resuspended, and analyzed by flow cytometry.

The percentage of cells in each phase was detected after staining with PI/RNase Staining Buffer. The cells were digested with EDTA-free trypsin, centrifuged at 1000 g for 10 min at 4 °C, resuspended with 1 mL of pre-cold PBS, and centrifuged at 1000 g and 4 °C. One milliliter of pre-cold 75% ethanol was added, followed by incubation at 4 °Covernight. After centrifugation at 1000 g for 10 min at 4 °C. Cells were collected, resuspended with 1 mL of pre-cold PBS, and centrifuged at 1000 g for 10 min at 4 °C. Cells were collected and gently resuspended with 500 μL of PI/RNase Staining Buffer, followed by incubation at 37°Cin dark. Red fluorescence was detected by flow cytometry with an excitation wavelength at 488 nm. The percentage of cells in different phases was analyzed with software.

### Statistical analysis

Statistical analysis was performed with SPSS version 20.0 (SPSS, Inc., Chicago, IL, USA). Data are expression as mean ± standard deviation (SD). Comparisons were done between groups with an unpaired Student’s t-test. A value of *P* < 0.05 was considered statistically significant.

## Results

### Histopathological features of the prostate in EAP rats

**Fig. 1:**
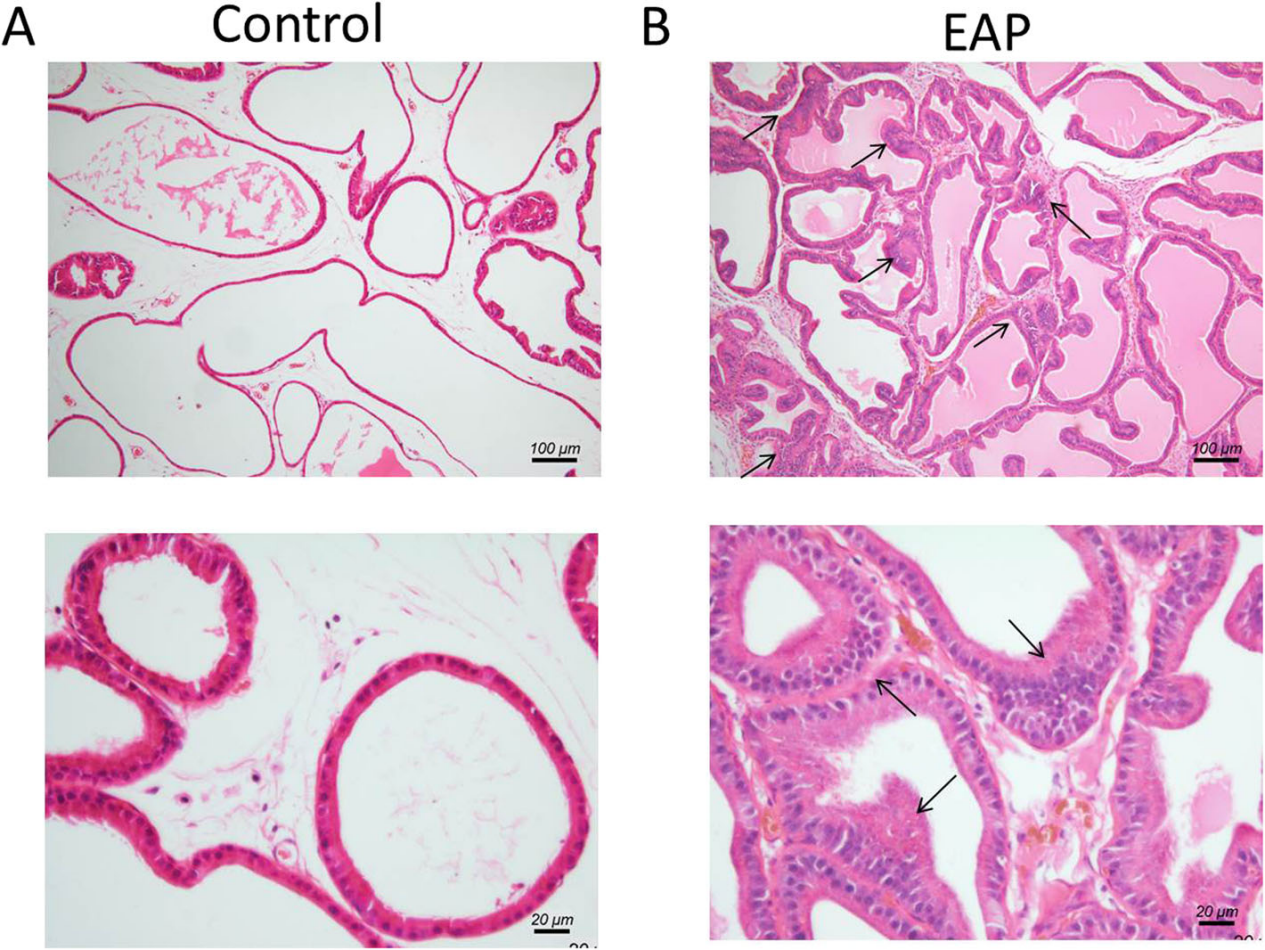
Histopathological features of ventral prostate tissues in two groups. In the normal rats, the glandular epithelium of the prostate wascomplete and clear without infiltration of inflammatory cells and hyperplasia (**a**). In the EAP rats, the prostate duct was irregular and showed diffuse and inhomogeneous hyperplasia; inflammatory cells infiltrated a par of basal lamina (**b**)

Histopathological features of rat ventral prostate are shown in Fig. 1. The prostatic glandular epithelium structure was complete and clear, and there were no inflammatory cells and hyperplasia in the normal rats (A). The prostate duct was irregular with diffuse and inhomogeneous hyperplasia, and inflammatory cells were observed in a part of basal lamina in the EAP rats (B). The histopathological features of the prostate in the EAP rats were consistent with the diagnostic criteria for human CP/CPPS [11].

### Effect of CP/CPPS on erectile function in rats

**Fig. 2:**
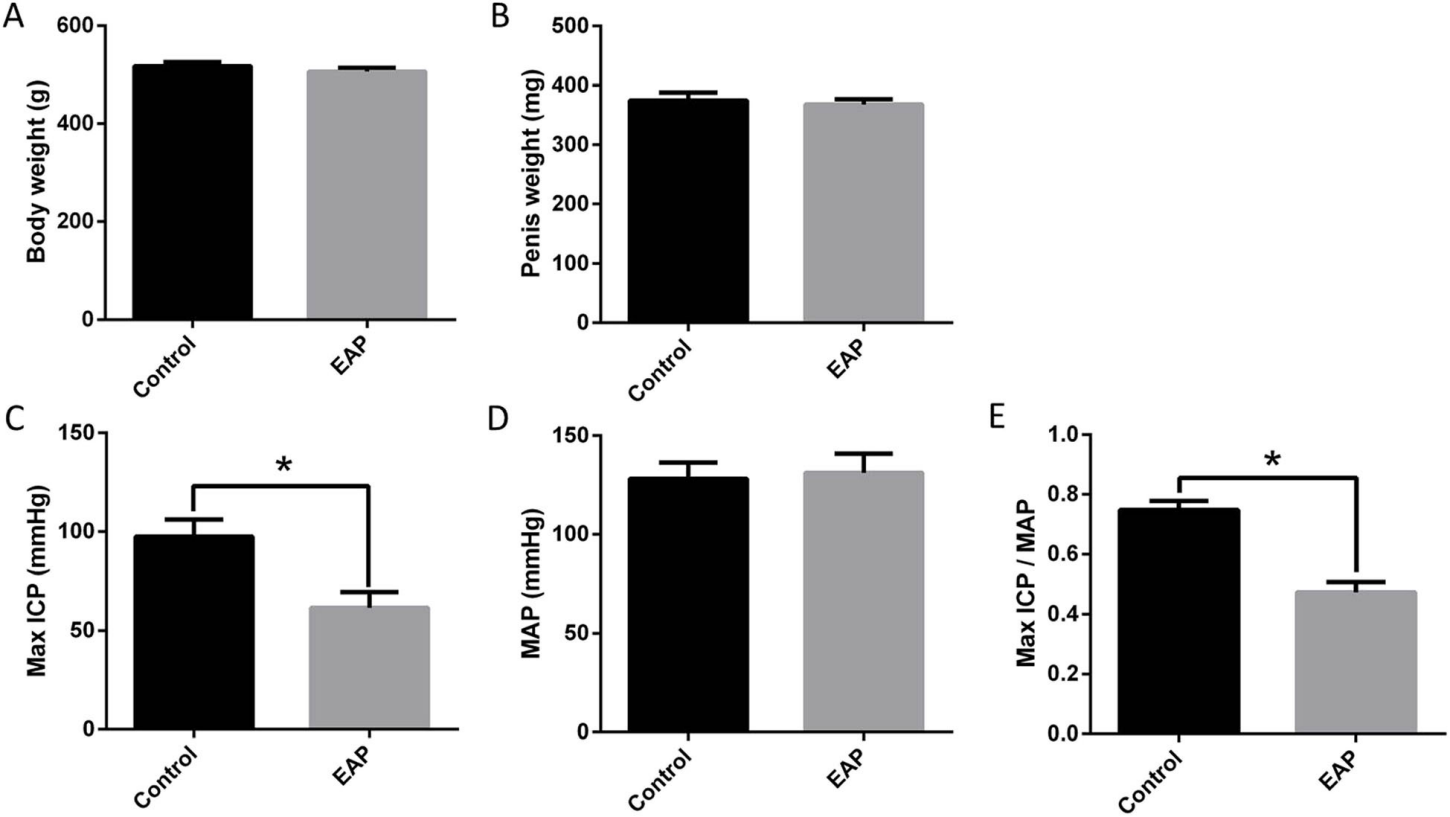
Body weight, penis weight and erectile function in two groups. There were no significant differences in the body weight (**a**) and penis weight (**b**) between normal control rats and EAP rats. The max ICP (**c**) and max ICP/ MAP ratio (E) were significantly lower in the EAP group than in the control group, MAP (**d**) was comparable between two groups

There were no significant differences in the body weight (517.12 ± 8.63 g vs 506.28 ± 7.98 g, A) and penis weight (374.71 ± 13.22 mg vs 368.05 ± 8.92 mg, B) between normal control rats and EAP rats. The max ICP (C) was significantly lower in the EAP group (61.60 ± 7.96 mmHg) than in the control group (97.40 ± 8.73 mmHg); the MAP (D) was comparable between two groups (128.02 ± 8.31 mmHg vs 131.23 ± 9.65 mmHg). The max ICP/ MAP ratio (E) was significantly lower in the EAP group (0.47 ± 0.03) than in the control group (0.75 ± 0.03) (Fig. 2).

### Inflammatory mediator and fibrosis of corpus cavernosum in EAP rats

**Fig. 3:**
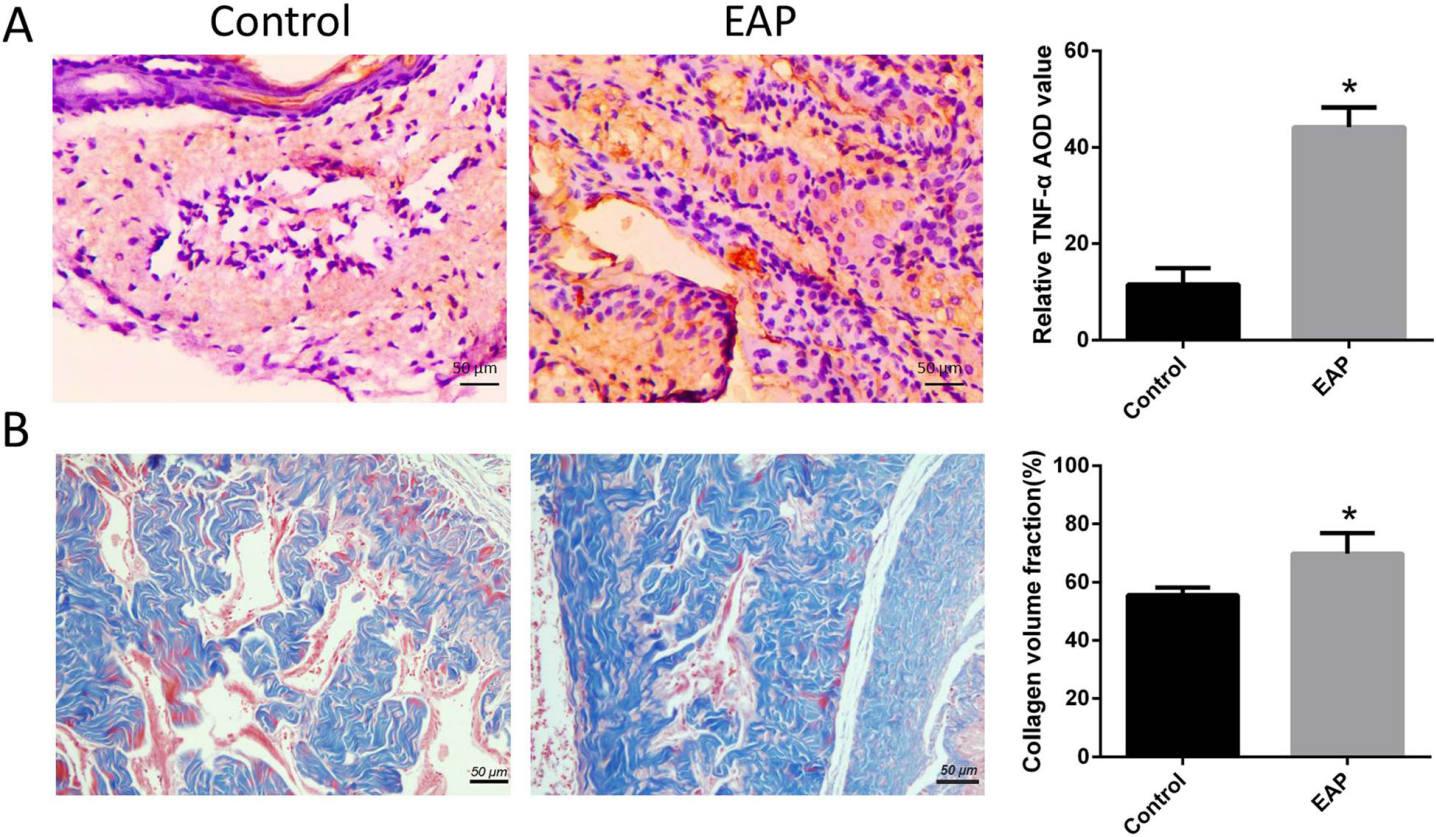
Increased TNF-α content and fibrosis in the corpus cavernosum of EAP rats. **a** The TNF-α content in the corpus cavernosum (right) of EAP rats (brown) was significantly higher than in the normal rats (left). **b** Collagen (blue) content in the corpus cavernosum (b right) of EAP rats was significantly higher than in the normal rats (b left), while the smooth muscle (red) significantly reduced in the corpus cavernosum

As showed in Fig. 3, immunohistochemistry (A) was done to detect the TNF-α expression. The TNF-α expression in the corpus cavernosum of EAP rats (right) was significantly higher than on normal rats (left). Masson’s staining (B) showed the collagen content in the corpus cavernosum of EAP rats (right) was significantly higher than in normal rats (left), but the corpus cavernosum smooth muscle significantly reduced in EAP rats.

### Isolation and culture of CCSMCs

**Fig. 4:**
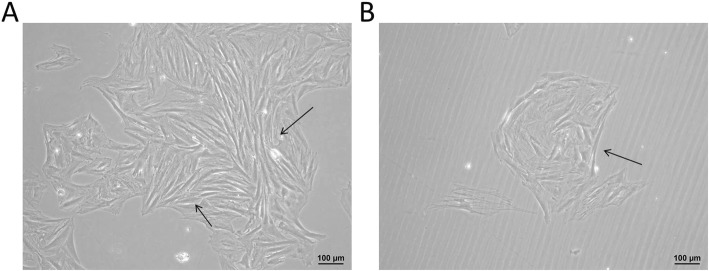
Isolation and culture of corpus cavernosum smooth muscle cells. With multiple differential adherence method, most cells were fusiform and arranged in bundles (**a**) and spirals (**b**)

The CCSMCs were isolated and purified. After incubation for 3 day, a few fusiform and astrocytes were found at the edge of tissue blocks. After multiple differential adherence methods, the smooth muscle cells gradually covered the dish within 14–22 d. As showed in Fig. 4, most of cells were fusiform and arranged in bundles (A) and spirals (B).

**Fig. 5:**
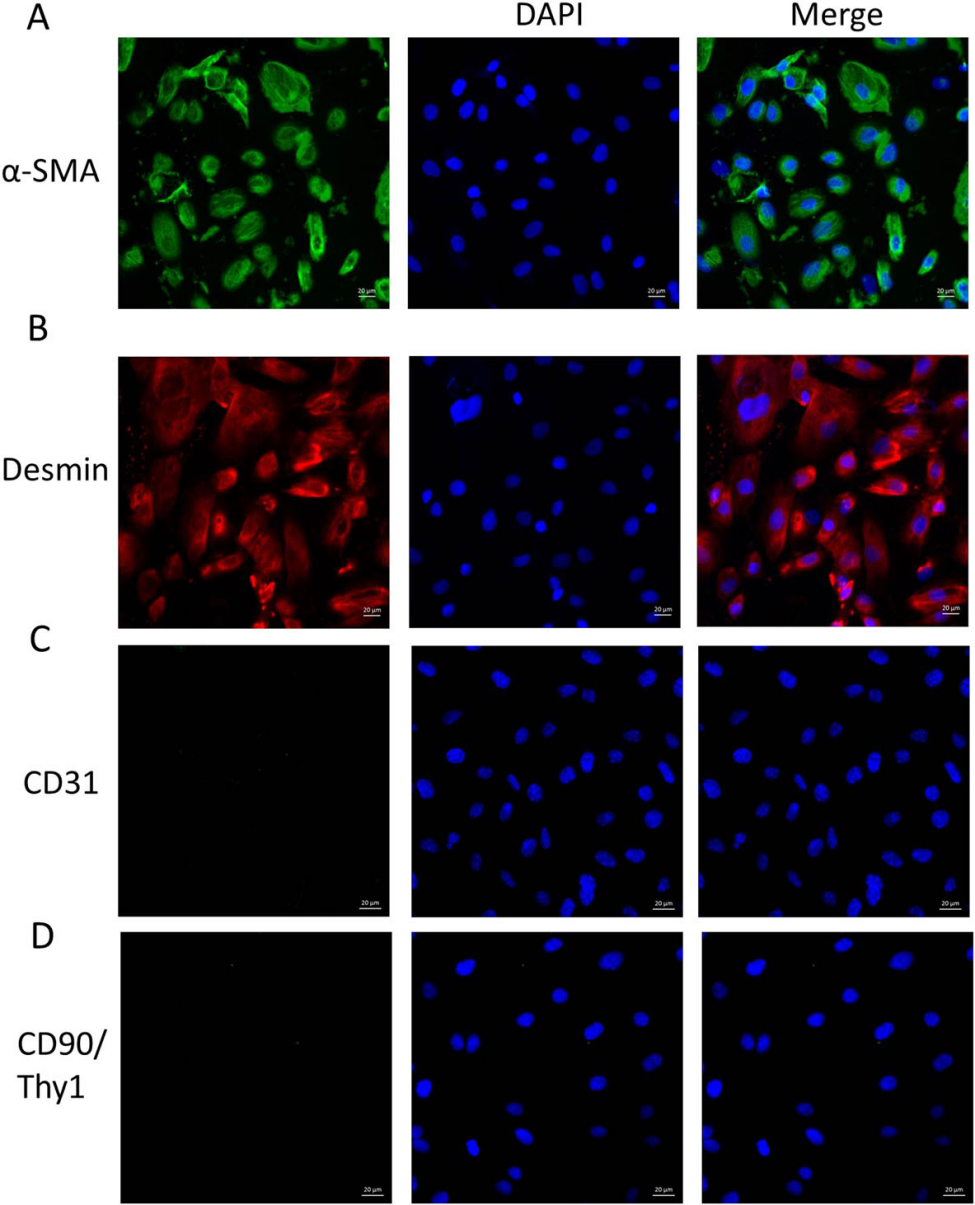
Identification of corpus cavernosum smooth muscle cells. Most cells expressed α-SMA (**a**) and Desmin (**b**), the cell nucleus was blue, and α-SMA and Desmin were mainly expressed in the cytoplasm and filamentous, which were in line with the localization of cytoskeletal proteins. Cells had no expression of CD31 (**c**) and CD90 / Thy1 (**d**), which were highly expressed in endothelial cells and fibroblast cells

### Identification of CCSMCs

As showed in Fig. 5, immunofluorescence staining for α-SMA and Desmin was done to identify the CCSMCs. Most cells express α-SMA (green, A) and Desmin (red, B), and the cell nuclei were blue. Both α-SMA and Desmin were mainly expressed in the cytoplasm and filamentous, which was in accordance with the protein localization of α-SMA and Desmin in the cytoskeleton. After multiple differential adherence, cells had no expression of CD31 (C) and CD90/Thy1 (D) which were highly expressed in the endothelial cells and fibroblast cells. Immunofluorescence staining confirmed that the CCSMCs had a high purity and could be used in subsequent experiments.

### Increased oxidative stress and reduced cGMP in the CCSMCs of EAP rats

In the EAP rat CCSMCs, the MDA content was 3.01 ± 0.07 nmol/mg prot, which was significantly higher than in the CCSMCs from normal control rats (2.75 ± 0.06 nmol/mg prot). The SOD activity in the EAP rat CCSMCs was 56.31 ± 2.23 U/mg prot, which was markedly lower than in the CCSMCs from normal control rats (SOD 78.83 ± 1.95 U/mg prot). The cGMP content was 8.85 ± 0.84 pmol/mg prot in the normal rat CCSMCs and 4.48 ± 0.62 pmol/mg prot in the CCSMCs from EAP rats, difference was statistically significant (Table 2).

**Table 2:** SOD activity, MDA content and cGMP content of CCSMCs from normal rats and EAP rats

Parameters	Control	EAP	*P*
SOD (U/mg prot)	78.83 ± 1.95	56.31 ± 2.23	0.001*
MDA (nmol/mg prot)	2.75 ± 0.06	3.01 ± 0.07	0.001*
cGMP (pmol/mg prot)	8.85 ± 0.84	4.48 ± 0.62	0.001*

*P < 0.05

### Calcium ion pathway disorder and phenotypic transformation of CCSMCs of EAP rat

**Fig. 6:**
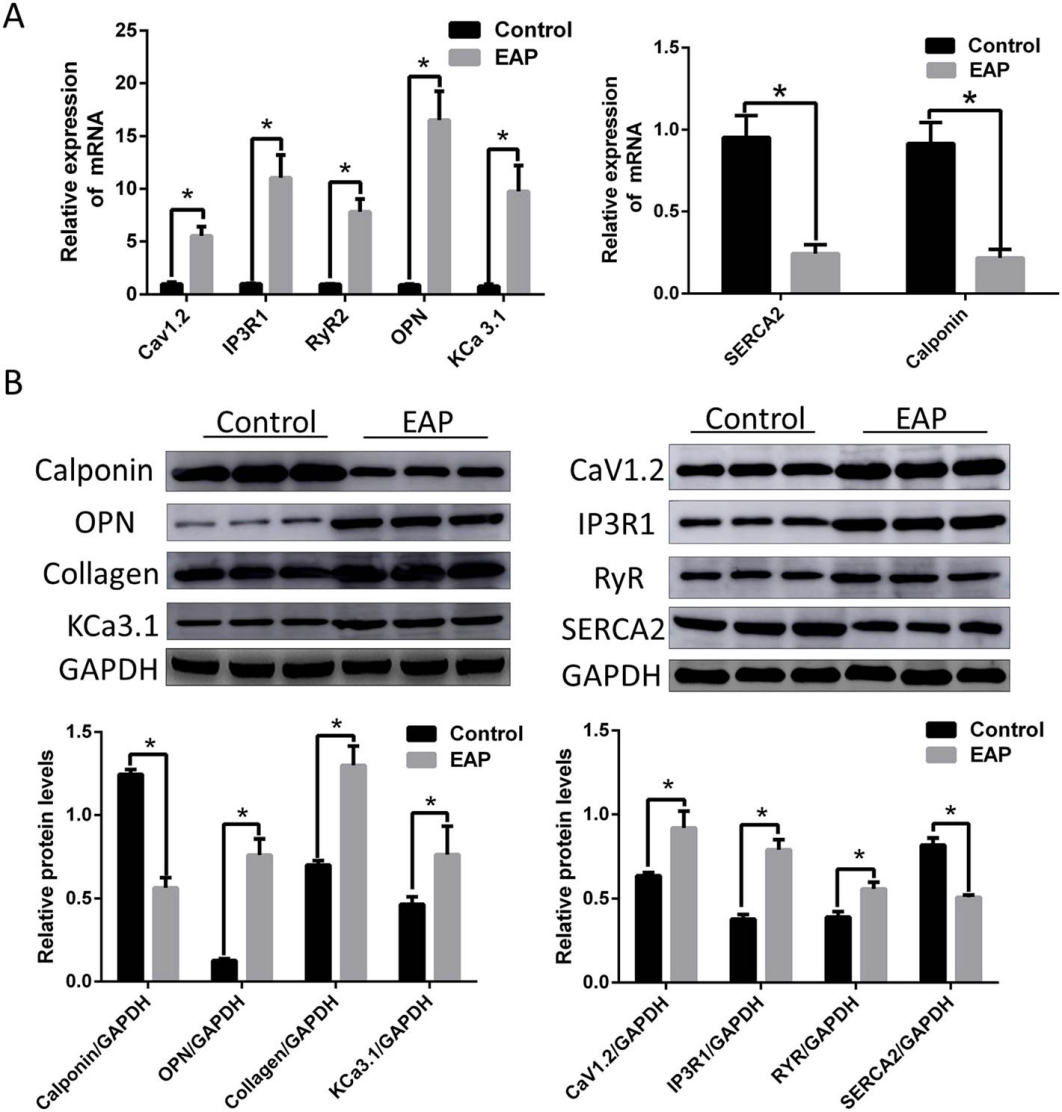
Calcium ion pathway disorder and phenotypic transformation of CCSMCs from EAP rats. **a** The mRNA expression of Calponin and SERCA2 decreased in the cavernous smooth muscle cells of EAP rats, and the mRNA expression of OPN, Cav1.2, IP3R1, RyR2 and KCa3.1 increased. **b** The protein expression of Calponin and SERCA2 decreased in the cavernous smooth muscle cells of EAP rats, and the protein expression OPN, Cav1.2, IP3R1, RyR2, KCa3.1 and collagen increased

As shown in Fig. 6, the mRNA (A) and protein (B) expression of CCSMC Cav1.2, IP3R1 and RyR2 significantly increased in the EAP rats while SERCA2 expression dramatically decreased in the EAP rats as compared to normal control rats. The mRNA and protein expression of Calponin significantly decreased while the expression of OPN, KCa3.1 and Collagen increased.

### Increased intracellular calcium and accelerated proliferation of CCSMCs from EAP rat

**Fig. 7:**
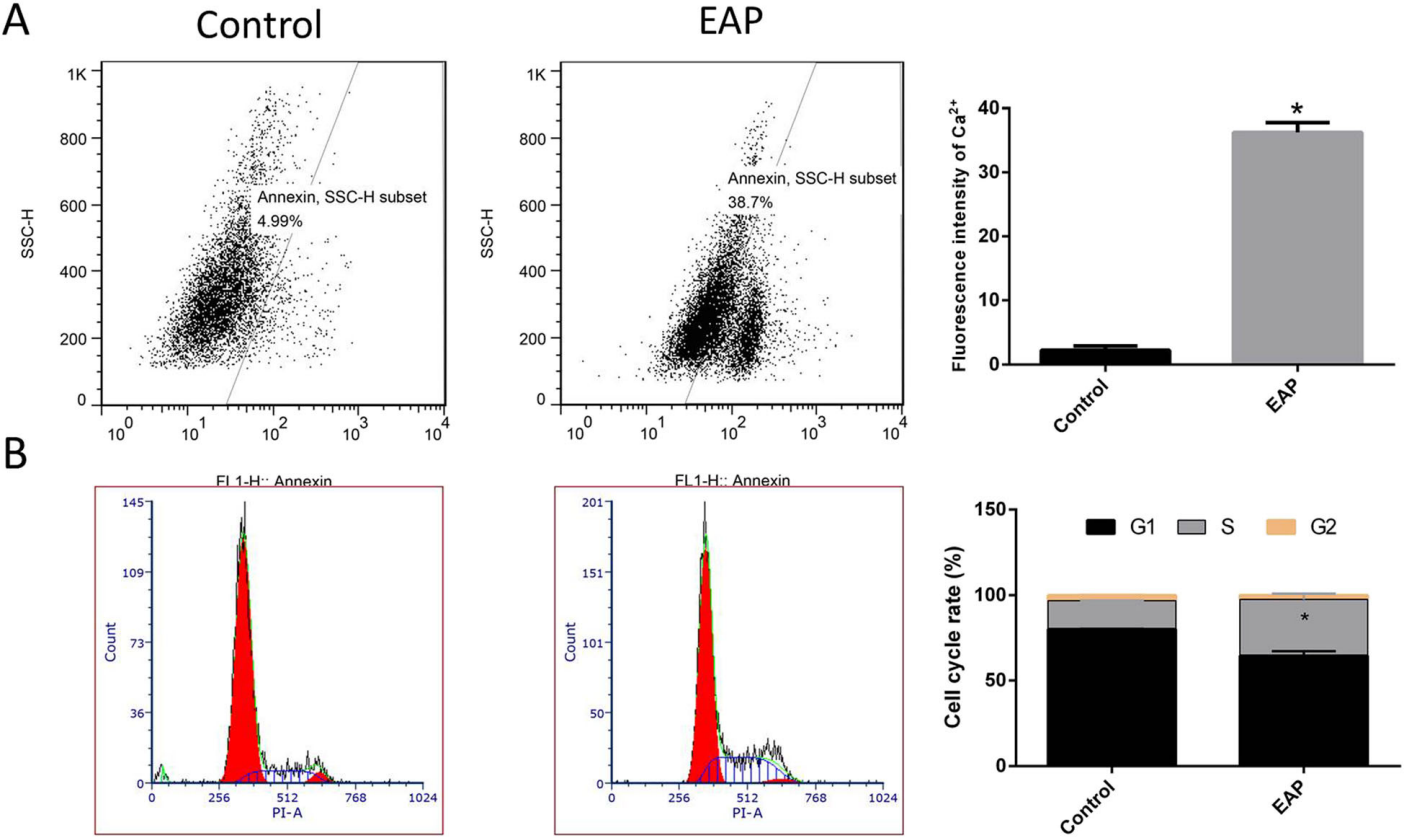
Increased intracellular calcium and cell proliferation of CCSMCs from EAP rats. **a** Flow cytometry showed the intracellular calcium of CCSMCs from EAP rats was significantly higher than in cells from normal rats. **b** The proportion of CCSMCs in the S phase from EAP rats increased significantly as compared to cells from normal rats

As shown in Fig. 7, flow cytometry showed the intracellular calcium in the CCSMCs from EAP rats was36.23 ± 0.89%, which was significantly higher than in the cells from normal rats (2.25 ± 0.38%) (A). Moreover, the proportion of CCSMCs in the S phase from EAP rats also increased significantly (B).

## Discussion

Available studies have shown that autoimmunity and oxidative stress play important roles in the pathogenesis of sexual dysfunction in CP/CPPS [5, 13]. Currently, little is known about the corpus cavernosum smooth muscle cells dysfunction secondary to chronic prostatitis. In the present study, a rat model of autoimmune prostatitis was successfully established, and rats CCSMCs were collected and purified with tissue block implantation and differential adherence method. Compared with normal rats, erectile function was compromised in the EAP rats; cytokine infiltration and collagen increased in the corpus cavernosum, the oxidative stress increased and the cGMP decreased in the CCSMCs from EAP rats. Furthermore, imbalance of intracellular calcium was also observed in the CCSMCs from EAP rats and the phenotype of CCSMCs from EAP rats was transformed from contractile to synthetic phenotype, which may be related to the fibrosis of corpus cavernosum. Our results indicate CP/CPPS-induced ED is associated with the imbalance of intracellular calcium and the phenotype transformation of CCSMCs.

Increased inflammatory mediators and oxidative stress have been reported in the CP/CPPS patients [3, 14]. TNF-α can increase monocyte-macrophages and activate NF-κB pathway to mediate inflammation cascade, reactive oxygen species production, and production of various adhesion molecules, such as ICAM-1, which promote the adhesion between neutrophils and smooth muscles, resulting in the basal layer damage. Eventually, foam cells form, leading to the atherosclerotic lesions in the penile vasculature [15, 16]. In addition, TNF-α can inhibit the expression of cGMP through NF-κB pathway [17], and decreased cGMP will inhibit the relaxation of smooth muscle cells [18].

SOD can specifically remove free radicals to relieve the injury due to free radical oxidation; MDA is the final metabolite of lipid oxidation and may reflect the degree of lipid oxidation [19]. The decreased SOD activity and increased MDA content were observed in the CCSMCs from EAP rats, the balance between oxidation and antioxidation was significantly disturbed, and the injuries induced by oxidation and lipoperoxidation increased.

In the smooth muscle cells, L-type calcium channels (CaV1.2) are the most common calcium channel in the prostate and vascular smooth muscle, and IP3R and RyR in the sarcoplasmic reticulum are also involved in the regulation of intracellular calcium [20]. Smooth muscle relaxation is mainly related to SERCA, which use the energy released by ATP hydrolysis to release calcium out of the cells, which plays an important role in maintaining intracellular calcium stability [21, 22]. Increased CaV1.2, IP3R and RyR and decreased SERCA were observed in the CCSMCs from EAP rats. The imbalance of intracellular calcium eventually leads to calcium overload in cells, causing excessive contraction of smooth muscle, which affects the perfusion of the corpus cavernosum and leads to ED [23]. Excessive Ca^2+^ can also activate the breaking down of phospholipids into fatty acids by the phospholipase A2 in the cell membranes, However, fatty acids can converted into a large amount of inflammatory mediators and free radicals, leading to cell and tissue damage [24].

Smooth muscle cells are highly malleable and can be phenotypically transformed under normal and pathological conditions to accommodate to microenvironment. Under physiological conditions, smooth muscle cells show a highly differentiated contractive phenotype, which mainly maintains the elasticity of blood vessels, and their proliferation and migration abilities are at a low level. Under pathological conditions such as inflammation, smooth muscle cells mainly display synthetic type, and their proliferation, migration and synthesis abilities are significantly improved, collagen secretion is increased to repair the injured tissues, but it is also a main factor resulting in arterial occlusion [25]. Calponin is an actin-binding protein in the cytoskeleton of smooth muscle cells and also a marker of contractile smooth muscle. OPN is a secreted acidic glycosylated phosphoprotein and a marker of synthetic smooth muscle cells. OPN plays important roles in regulating the adhesion and migration of vascular smooth muscle cells [26]. In the CCSMCs from EAP rats, the Calponin expression decreased, the OPN expression increased and the proportion of cells in S phase increased, indicating that the proliferation of smooth muscle cells was improved and CCSMCs are transformed from contractile to synthetic phenotype. Under this condition, the collagen synthesis increases, leading to the fibrosis of corpus cavernosum.

Elevated intracellular calcium may activate KCa3.1 (a calcium-activated potassium channel) and cause non-voltage-dependent membrane hyperpolarization, which drive the Ca^2+^ influx. KCa3.1 expression also significantly increased in the CCSMCs from EAP rats. With further increase in the intracellular calcium, KCa3.1 channel opening due to the persistent smooth muscle membrane potential hyperpolarization is the primary manifestation of cell proliferation and migration, and KCa 3.1 plays a leading role in vascular smooth muscle hyperplasia [27]. TRAM-34 [28], a KCa3.1 specific inhibitor, can reduce KCa3.1 expression and significantly inhibit the transformation of contractile smooth muscle to synthetic smooth muscle [29]. Thus, TRAM-34 may become a new treatments forED in CP/CPPS.

## Conclusion

**Fig. 8:**
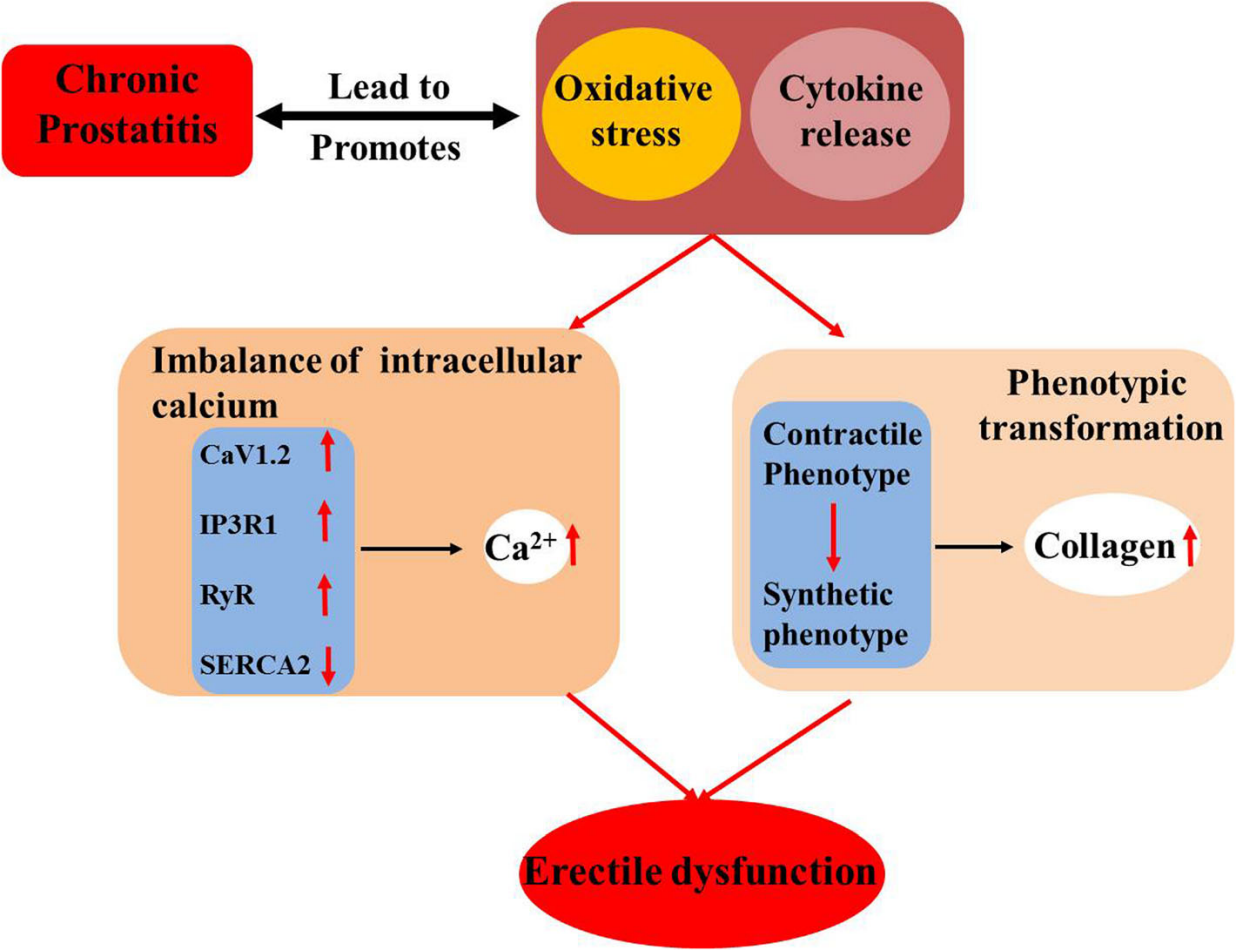
Chronic prostatitis promotes cytokine production in the corpus cavernosum and increases oxidative stress, resulting in imbalance of intracellular calcium in CCSMCs. Moreover, CCSMCs are transformed from contractile to synthetic phenotype, and the collagen synthesis increases, resulting in the fibrosis of the corpus cavernosum

Chronic prostatitis promotes the infiltration of inflammatory cells into the corpus cavernosum and increases oxidative stress, resulting in imbalance of intracellular calcium in the CCSMCs. Moreover, CCSMCs are transformed from contractile to synthetic phenotype, and the collagen synthesis increases causing the fibrosis of the corpus cavernosum (Fig. 8). This may be one of important mechanisms underlying the pathogenesis of ED in the EAP rats. More studies are needed to confirm our findings.

## Data Availability

Please contact author for data requests.

## Abbreviations

CP/CPPS: Chronic prostatitis/chronic pelvic pain syndrome
ED: Erectile function
EAP: Experimental autoimmune prostatitis
PTHS: Prostate tissue homogenate supernatant
CCSMCs: Corpus cavernosum smooth muscle cells
NO: Nitric oxide
eNOS: Endothelial nitric oxide synthase
nNOS: Neuronal nitric oxide synthase
cGMP: Cyclic guanosine monophosphate
SD: Sprague-Dawley rats
ICP: Intracavernosal pressure
MAP: Mean arterial pressure
SOD: Superoxide Dismutase
MDA: Malondialdehyde
OPN: Osteopontin
CaV: The cardiac L-type calcium channel
RyR: Ryanodine receptor
IP3R: Inositol-1,4,5-trisphosphate receptor
KCa: The Ca^2+^-activated K^+^ channel
SERCA: Sarco-endoplasmic reticulum Ca^2+^-ATPase
